# Metabolic Targets of Coenzyme Q10 in Mitochondria

**DOI:** 10.3390/antiox10040520

**Published:** 2021-03-26

**Authors:** Agustín Hidalgo-Gutiérrez, Pilar González-García, María Elena Díaz-Casado, Eliana Barriocanal-Casado, Sergio López-Herrador, Catarina M. Quinzii, Luis C. López

**Affiliations:** 1 Departamento de Fisiología, Facultad de Medicina, Universidad de Granada, 18016 Granada, Spain; pgonzalez@ugr.es (P.G.-G.); elenadiaz@ugr.es (M.E.D.-C.); elianabc@ugr.es (E.B.-C.); sergiolopezhe@correo.ugr.es (S.L.-H.); 2 Centro de Investigación Biomédica, Instituto de Biotecnología, Universidad de Granada, 18016 Granada, Spain; 3 Department of Neurology, Columbia University Medical Center, New York, NY 10032, USA; cmq2101@cumc.columbia.edu

**Keywords:** coenzyme Q10, ubiquinone-10, ubiquinol-10, mitochondria, OxPhos, super-complexes, sulfide metabolism, one-carbon metabolism, proline metabolism

## Abstract

Coenzyme Q10 (CoQ_10_) is classically viewed as an important endogenous antioxidant and key component of the mitochondrial respiratory chain. For this second function, CoQ molecules seem to be dynamically segmented in a pool attached and engulfed by the super-complexes I + III, and a free pool available for complex II or any other mitochondrial enzyme that uses CoQ as a cofactor. This CoQ-free pool is, therefore, used by enzymes that link the mitochondrial respiratory chain to other pathways, such as the pyrimidine de novo biosynthesis, fatty acid β-oxidation and amino acid catabolism, glycine metabolism, proline, glyoxylate and arginine metabolism, and sulfide oxidation metabolism. Some of these mitochondrial pathways are also connected to metabolic pathways in other compartments of the cell and, consequently, CoQ could indirectly modulate metabolic pathways located outside the mitochondria. Thus, we review the most relevant findings in all these metabolic functions of CoQ and their relations with the pathomechanisms of some metabolic diseases, highlighting some future perspectives and potential therapeutic implications.

## 1. Introduction

Ubiquinone or coenzyme Q (CoQ) is an endogenously synthesized vital molecule that is present in many unicellular and pluricellular organisms, the latter being mostly localized in the mitochondria. In mammalian cells, at least 14 proteins are needed to synthetize CoQ, and its biosynthesis starts with the formation of a 4-hydroxybenzoic acid (4-HB) head group and a lipophilic polyisoprenoid tail. Whereas the 4-HB is derived from tyrosine or phenylalanine, the polyisoprenoid tail is produced by addition of isopentenyl diphosphate molecules, derived from the mevalonate pathway, to farnesyl diphosphate or geranylgeranyl diphosphate in multiple steps catalyzed by the polyprenil diphosphate synthase. In human and mice, this enzyme is a heterotetramer and the two subunits are encoded by *PDSS1* and *PDSS2*. Another enzyme, encoded by *COQ2*, mediates the conjugation of the benzoquinone ring to the side chain; while four others, encoded by *COQ3*, *COQ5*, *COQ6* and *COQ7*, reside in the mitochondrial inner membrane and modify the benzoquinone ring of CoQ by reactions of methylation, decarboxylation and hydroxylation [1]. Moreover, four other proteins, encoded by *COQ4*, *COQ8A*, *COQ8B* and *COQ9*, seem to have different regulatory functions over other CoQ biosynthetic proteins [1]. In particular, COQ9 physically interacts with COQ7 and has the ability to bind some CoQ biosynthetic intermediates, including demethoxyubiquinone (DMQ), the substrate for COQ7 [2,3]. Furthermore, some other proteins (ADCK1, ADCK2, ADCK5 and PTC7p), may have the capacity to increase/reduce the CoQ biosynthetic rate by phosphorylating/dephosphorylating some CoQ biosynthetic proteins, e.g., COQ7, or by other unknown mechanisms [1]. However, the CoQ biosynthesis in mitochondria is not fully understood. Interestingly, Golgi has the COQ2 homolog UBIAD1 [4], which also supports the biosynthesis of CoQ also in this organelle.

Mutations in any of the genes involved in CoQ_10_ biosynthesis cause primary CoQ_10_ deficiency, a mitochondrial syndrome associated with clinically heterogeneous diseases. Specifically, five major phenotypes have been described: (1) encephalomyopathy (recurrent myoglobinuria, encephalopathy and mitochondrial myopathy), (2) cerebellar ataxia (cerebellar atrophy associated with other neurologic manifestations and, occasionally, endocrine dysfunctions), (3) infantile multisystemic form, (4) isolated myopathy, characterized by muscle weakness, myoglobinuria, exercise intolerance and elevated creatine kinase (CK), and (5) nephropathy. Hypertrophic cardiomyopathy, retinopathy or optic atrophy, and sensorineural hearing loss have been also reported in some patients [5]. Additionally, CoQ_10_ deficiency has been identified as a secondary consequence of other medical conditions. This category includes patients with mutations in the aprataxin (APTX) gene, causing ataxia and oculomotor apraxia, with mutations in the electron-transferring flavoprotein dehydrogenase gene (ETFDH), causing isolated myopathy, and with mutations in Serine/threonine-protein kinase B-raf (BRAF), causing cardiofaciocutaneous (CFC) syndrome [6]. Moreover, CoQ_10_ deficiency has been reported in association with pathogenic mitochondrial DNA (mtDNA) depletion, deletions, or point mutations [6]. Also, a decrease in the levels of CoQ biosynthetic proteins has been related to secondary CoQ deficiency in various mouse models of mitochondrial diseases [7], as well as in muscle and adipose tissue of patients and a mouse model with insulin resistance [8]. The wide clinical spectrum of CoQ_10_ deficiencies must be related to its structure, characteristics and multiple metabolic functions.

Structurally, CoQ is differentiated into a benzoquinone ring, which confers the redox properties of the molecule, and a polyprenoid tail, which is responsible for its lipophilicity [1,9,10]. The benzoquinone ring may exist in three redox states, fully oxidized (producing ubiquinone), semiquinone radical (producing ubisemiquinone) and fully reduced (producing ubiquinol). Also, the polyprenoid tail may be different in size, which is a characteristic of the different CoQ forms present in different organisms. This size seems to be determined by the homolog of the human PDSS1 protein in different species. For instance, the polyprenoid tail of the CoQ in *Saccharomyces cerevisiae* has six isoprenoid units, leading to the production of Coenzyme Q6 (CoQ_6_), mouse has a major form of nine isoprenoid units and a minor form of ten isoprenoid units, leading to the production of Coenzyme Q9 (CoQ_9_) and Coenzyme Q10 (CoQ_10_) respectively, and in human, CoQ_10_ is the major form, and CoQ_9_ is the minor one [1]. The distribution of CoQ_9_ and CoQ_10_ in mouse and human varies between tissues and cell types, but specific functional differences between both CoQ forms have not been described as yet [1].

The lipophilicity and the redox capability provide CoQ with the characteristics to perform most of its functions. Generally, CoQ is considered as one of the most important endogenous antioxidants, being especially effective in reducing/preventing lipid peroxidation. This antioxidant role of CoQ has been confirmed by (1) studies of characterization of in vitro and in vivo models of CoQ deficiency, which show increased generation of reactive oxygen species (ROS) and oxidative damage, pointing out CoQ’s role in ROS generation and scavenging, (2) studies of CoQ_10_ supplementation in the same models of CoQ deficiency, showing that the rescue of biochemical and clinical phenotypes and survival correlate with improvement of oxidative stress, demonstrating the antioxidant effects of CoQ_10_, and (3) studies in different models of increased oxidative stress in which CoQ_10_ is able to reduce markers of oxidative damage [2,11,12,13,14,15,16]. The antioxidant capacity of CoQ is due to its capability to directly reduce ROS, but also to regenerate other antioxidants, e.g., tocopherol and ascorbate [17]. To work as an antioxidant, CoQ must be able to reduce itself after being oxidized. This can be done by different NAD(P)H oxidoreductases localized in the plasma membrane, such as NADH-cytochrome b5 reductase, NAD(P)H:quinone oxidoreductase 1, or NADPH-CoQ reductase [17]. For all these functions in cell membranes, CoQ must be distributed among them and that distribution seems to be regulated by specific proteins [18,19].

**Figure 1:**
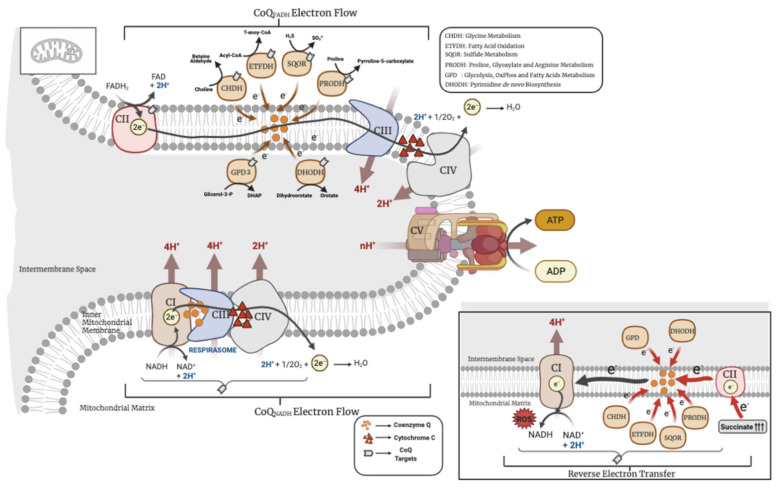
Metabolic uses of coenzyme Q (CoQ) in mitochondria. CHDH = choline dehydrogenase; ETFDH = electron transfer flavoprotein dehydrogenase; SQOR = sulfide:quinone oxidoreductase; PRODH = proline dehydrogenase; GPD2 = glycerol phosphate dehydrogenase; DHODH = dihydroorotate dehydrogenase. The above image represents the forward electron transport and the image below, the reverse electron transport (RET).

In addition to this antioxidant capacity and redox regulation, which have been extensively reviewed in the literature, CoQ has other key metabolic functions in mitochondria, where its main biosynthetic process mostly occurs. Thus, this review is focused in describing the functional metabolic roles of CoQ in mitochondria and how, through those functions, CoQ can also influence other metabolic pathways outside of mitochondria. Particularly, this review provides an in-depth look at the metabolic pathways linked to a functional structure called the CoQ-junction (Figure 1). As we will emphasize, all these processes have important physiological and pathophysiological implications.

## 2. CoQ in the OxPhos System

CoQ was first isolated by Professor Frederick Crane in 1957 in beef heart, describing the presence and function of CoQ in the mitochondrial respiratory chain. Since then, its proprieties as a mobile electron carrier in the mitochondrial respiratory chain and as a molecule with redox capabilities in the cell have been widely studied [20]. In mitochondria, CoQ is mainly localized in the inner mitochondrial membrane, where it accepts electrons from NADH through NADH ubiquinone oxidoreductase (CI), and/or from FADH_2_ through succinate dehydrogenase (CII). Those electrons are then transferred to cytochrome c through CoQH_2_-cytochrome c reductase (CIII), and the cytochrome c transfers the electrons to the oxygen through cytochrome c oxidase (CIV). The transfer of electrons among the complexes is accompanied by the pumping of protons to the intermembrane space, generating a proton motive force that is used by the ATP synthase (CV) to produce the ATP. The transfer of electrons in the mitochondrial respiratory complexes is favored by the formation of super-complexes, a supramolecular organization that joins the mitochondrial individual complexes in one single structure, in which CoQ is an essential component [21]. Therefore, the complexes can be found individually or, varying in the number of complexes, organized in super-complexes, which encompass complexes I, III and IV. CII, however, has not been proven to be in association with any other complex [22,23]. Both individual complexes and super-complexes are functional, indicating that the organization in super-complexes has to provide some advantages over the individual complexes, i.e., better stability, lower ROS production, or a better mobilization of the electron transfer from the mobile carriers, CoQ or cytochrome c, to their targets [21]. For years, CoQ was thought to be free in the mitochondrial membranes in a homogeneous pool ready to be used by any enzyme that needed it [24]. However, more recently, it has been demonstrated that the supramolecular organization of complexes in super-complexes dynamically segments the CoQ molecules in: (1) a pool attached and engulfed by the super-complexes I + III, exclusively dedicated to the oxidation of NADH (CoQ_NADH_ pool), and (2) a free pool, available for CII or any other enzyme that uses CoQ as a cofactor (CoQ_FADH_ pool) [25]. However, the two pools can interact with each other. For example, fasting leads to an energy shift from glucose to fatty acids, which results in a decrease in the NADH/FADH_2_ ratio. In that situation, the CoQ_FADH_ pool can be over-reduced and the electron flux could be reversed in order to reduce NAD^+^ through the CoQ_NADH_ pool, thus restoring the oxidized CoQ [25]. This reverse electron transport (RET) from CoQ to NAD^+^ enhances the scape of electrons and produces an accumulation of ROS in the form of anion superoxide at the level of CI, resulting in CI-derived oxidative damage [25,26]. The CI-derived oxidative damage leads to the degradation of CI with the subsequent release of CIII and CoQ_NADH_ pool from the super-complexes. This partial release of the CoQ_NADH_ pool is then used to restore the oversaturated CoQ_FADH_ pool by the shift in the metabolism from carbohydrates to fats [25]. In addition, the generation of the RET could be enhanced by the electrons that other enzymes transfer to CoQ, such as glycerol-3-phosphate dehydrogenase or the electron-transferring flavoprotein, also leading to ROS production [16,26]. Therefore, both the direction of the flow of electrons in the mitochondrial respiration and the formation of mitochondrial complexes/super-complexes are modulated by the CoQH_2_/CoQ ratio, which serves as a sensor to the metabolic status of the mitochondria [27]. This key role of the CoQH_2_/CoQ ratio has been experimentally demonstrated with the uses of the alternative oxidase (AOX), which accepts electrons from CoQ and, therefore, contributes to decrease the CoQH_2_/CoQ ratio, resulting in a decline of RET and ROS production [28]. However, it is important to highlight that the ROS derived from RET may provide some beneficial effects since they can work as cellular signalers. The toxic or beneficial effect of ROS depends on their amount, localization and type of ROS [29]. In fact, ROS production via RET at CI, controlled by the CoQH_2_/CoQ ratio, has been associated with different physiological processes, such as: (1) the in vitro differentiation of myoblasts into myotubes [30], (2) the metabolic shift from carbohydrates to fatty acids [27], (3) the macrophages’ reprograming and activation under bacterial stimuli [31], (4) the sensing of oxygen levels by the specialized cells in the carotid body [32], (5) the increase of the lifespan in *Drosophila melanogaster* [33] or (6) in the oxidative damage produced by the reperfusion of heart or brain after infarction or stroke, since the interruption of electron flow induces the accumulation of succinate in the ischemic phase of these conditions, follow by a rapid use of that succinate in the reperfusion phase, leading to an increase of the CoQH_2_/CoQ ratio and RET [34,35]. This underlies the importance of the CoQH_2_/CoQ ratio and RET in both physiological and pathophysiological contexts.

From a different perspective, the importance of CoQ in the mitochondrial respiratory chain is evidenced by the OxPhos defect and decreased ATP production in tissues from patients with primary CoQ_10_ deficiency, as well as in cells and animal models of CoQ deficiency [5]. For instance, decreased activities of CoQ-dependent mitochondrial complexes have been described in muscle and/or skin fibroblasts from the first patients with identified molecular defects in the CoQ biosynthetic pathway, i.e., *COQ2*, *PDSS2*, *PDSS1* or *COQ9* [36,37,38,39,40]. Curiously, the severity of the OxPhos defect inversely correlates with ROS production and oxidative damage in skin fibroblasts from patients with primary CoQ_10_ deficiency [12,13]. This correlates with the low mitochondrial ROS production in the cardiac mitochondria from the Coq7 conditional Knockout (KO) mice, which contains only 10% of the normal CoQ levels [41]. The connection between CoQ levels and bioenergetics defects has also been demonstrated in conditions of pharmacological inhibition of CoQ biosynthesis in human skin fibroblasts and neurons [15]. Also, mouse embryonic fibroblasts (MEFs) from a *Coq7* knockout mouse model or a *Coq9* knock-in mouse model (*Coq9^R239X^*), generated by independent groups, show a reduction in mitochondrial respiration and a decrease in ATP production [42,43,44,45]. COQ7 is a hydroxylase that uses demethoxyubiquinone (DMQ) as substrate in the CoQ biosynthetic pathway, and it needs COQ9 for its stability and function [2,3,44]. Consequently, *Coq7* knockout mice and *Coq9^R239X^* mice accumulate DMQ, which could compete with CoQ and inhibit the CI + III activity [46]. Additionally, the mouse model of CoQ deficiency due to a spontaneous mutation in *Pdss2* (*Pdss2^kd/kd^*) shows decreased activities of complex I and II + III activities in kidneys, the most clinically affected tissue [47]. Interestingly, OxPhos defects in cell and animal models of CoQ deficiency are not always rescued by CoQ_10_ supplementation, and their role in the pathogenesis of the disease seems to be variable, probably depending on the molecular defect and/or tissue specificity. In vitro, CoQ_10_ supplementation for 7 days normalizes the ATP levels and ATP/ADP ratio in skin fibroblast from patients with mutations in *PDSS2, COQ2* and *COQ9* [48]. However, other short-tail ubiquinone analogs are not able to restore the ATP synthesis, demonstrating the importance of the CoQ structure in the function of the OxPhos system [48]. In vivo, the low absorption and bioavailability of the exogenous CoQ_10_ limits its bioenergetic effects in some tissues. In *Pdss2^kd/kd^* mice, CoQ_10_ supplementation does not rescue the CoQ-dependent complexes activities in kidneys, although therapeutic benefits were reported, presumably due to other CoQ_10_ functions [47,49]. In *Coq9^R239X^* mice, ubiquinol-10 (CoQ_10_H_2_), but not ubiquinone-10 (CoQ_10_), is able to partially increase CI + III activity in the brain due to its superior absorption, bioavailability and mitochondrial uptake [50], leading to an increase in survival [51]. Since *Coq9^R239X^* mice accumulates DMQ, the reduction in the levels of DMQ achieved by the treatment with beta-resorcylic acid (β-RA) is able to increase the bioenergetics and obtain therapeutic outcomes that are superior to those obtained under CoQ_10_H_2_ supplementation [51]. The decrease on the levels of DMQ, and its subsequent therapeutic benefits, is also achieved by b-RA in the conditional knockout *Coq7* mouse model, as well as in skin fibroblasts from patients with mutations in *COQ7* or *COQ9* [41,52,53], pointing out the structural specificity of CoQ in its function in the mitochondrial respiratory chain. The importance of CoQ in the OxPhos system has also been revealed in cases of secondary CoQ deficiencies, although, in those cases, it is unclear the contribution of the different functions of CoQ to the pathophysiologic characteristics of the disease.

## 3. The Role of CoQ in the Regulation of Sulfide Metabolism and Others Linked Pathways

**Figure 2:**
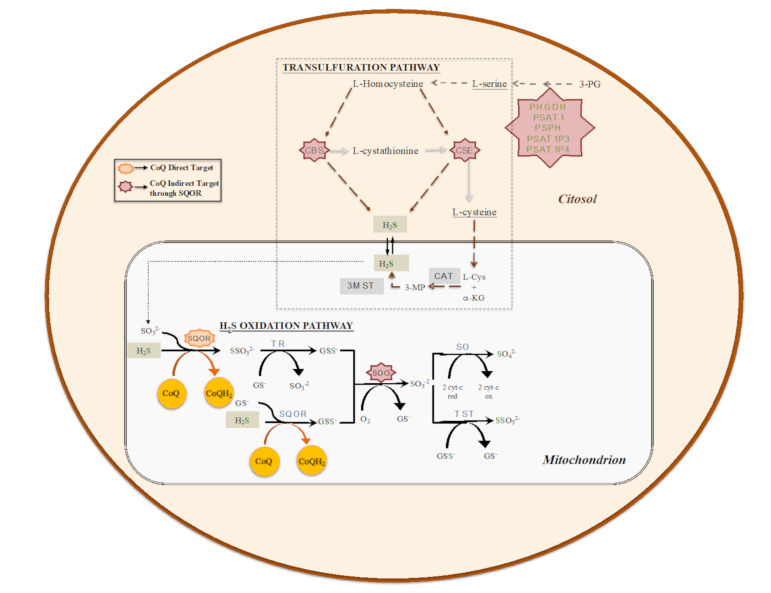
The biosynthetic (transsulfuration) and catabolic (oxidation) pathways of H_2_S. Transsulfuration pathway involves the enzymes cystathionine β-synthase (CBS), cystathionine γ-lyase (CSE) and pyridoxal-5′-phosphate (PLP)-independent 3-mercaptopyruvate sulfurtransferase (3MST). Mitochondrial H_2_S oxidation pathway involves the enzymes sulfide:quinone oxidoreductase (SQOR), sulfur dioxygenase (SDO; also known as ETHE1 or persulfide dioxygenase), sulfite oxidase (SO), thiosulfate sulfurtransferase or rhodanese (TST) and thiosulfate reductase (TR).

Given its central role in the mitochondrial respiratory chain, CoQ links the mitochondrial respiratory chain to other mitochondrial enzymes and pathways in the functional structure called the CoQ-junction. One of these enzymes is the sulfide:quinone oxidoreductase (SQOR), which catalyzes the first step in the mitochondrial sulfide oxidation pathway. SQOR couples H_2_S oxidation to CoQ reduction, forming a protein-bound persulfide (Figure 2). The SQOR-bound persulfide is transferred to a sulfane sulfur acceptor, e.g., such as glutathione (GSH) or sulfite, resulting in the generation of GSH persulfide (GSSH) or thiosulfate, respectively. The persulfide group from GSSH is further oxidized to sulfite by an iron-dependent sulfur dioxygenase (SDO) (also known as ETHE1 or persulfide dioxygenase). Sulfite can then either be oxidized to sulfate by sulfite oxidase (SO) or converted to thiosulfate via addition of a persulfide catalyzed by the thiosulfate sulfurtransferase or rhodanese (TST). The sulfane sulfur from thiosulfate can be remobilized by another sulfurtransferase called thiosulfate reductase (TR) (Figure 2) [54,55,56,57]. The H_2_S used as a substrate by the SQOR is produced by at least three enzymes, of which two are in the transsulfuration pathway (Figure 2), localized in the cytosol. These two enzymes are Cystathionine β-synthase (CBS), which produces H_2_S primarily by condensation of cysteine and homocysteine, and cystathionine γ-lyase (CSE), which produces H_2_S primarily by α- and β-elimination of cysteine, generating pyruvate and ammonia. A third enzyme, 3-mercaptopyruvate sulfurtransferase (3MST) [56], produces H_2_S from 3-mercaptopyruvate (3MP), an achiral α-keto acid generated by cysteine aminotransferase (CAT) from L-cysteine and α-ketoglutarate (α-KG). The CAT/3MST pathway has also been detected in mitochondria, which opens the possibility of an intramitochondrial production of H_2_S (Figure 2) [58].

**Figure 3:**
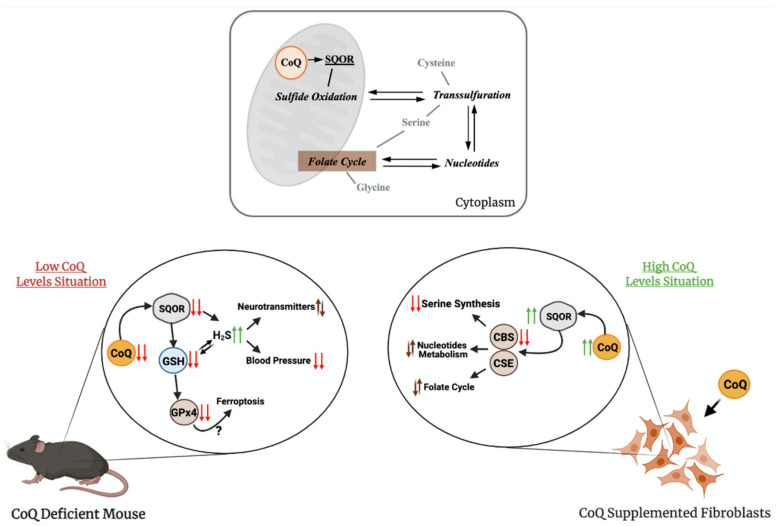
The relation of CoQ with sulfide metabolism and other linked pathways. The upper panel shows how CoQ is connected to sulfide metabolism and one-carbon metabolism. The bottom panel shows the main finding on these pathways in conditions of supraphysiological levels of CoQ_10_ or deficiency in CoQ_10_. SQOR = sulfide:quinone oxidoreductase; CBS = cystathionine-β-synthase; CSE = cystathionine γ-lyase; GSH = glutathione; H_2_S = hydrogen sulfide; GPx4 = glutathione peroxidase 4.

The regulation of production and the use of H_2_S occurs at the level of expression and activity of the biosynthetic and catabolic enzymes. For example, tonic inhibition of CBS is relieved during hypoxia, leading to elevated H_2_S production [59], the restriction in sulfur aminoacids (SAAs) upregulates the transsulfuration pathway, increasing H_2_S production [60,61], or the levels of SQOR are increased under hypoxia and restriction in SAAs [62,63]. However, an integration of regulatory aspects in both the biogenesis and the catabolism of H_2_S has not been clearly formulated. Recently, our group has demonstrated that CoQ_10_ supplementation leads to an upregulation of SQOR, together with a downregulation of CBS and CSE, in vitro (Figure 3) [64].

Therefore, CoQ_10_ regulates both pathways in opposite directions by modifying the gene expression and protein levels of SQOR, CBS and CSE. Although the mechanisms of this regulation have not been identified yet, these results may have relevant therapeutic implications for diseases with disrupted H_2_S metabolism. For instance, the reduction in the H_2_S levels, as a consequence of the effects of CoQ_10_ supplementation, could be a therapeutic approach in genetic diseases with defects in sulfide metabolism enzymes, i.e., the ethylmalonic encephalopathy caused by mutations in *ETHE1* [65], or the Leigh syndrome caused by mutations in *SQOR* [66]. In both cases, the accumulation of high levels of H_2_S induces mitochondrial toxicity and, consequently, the repression of the CBS and CSE, together with the stimulation of SQOR, may limit that toxicity by reducing the levels of H_2_S. CoQ_10_ supplementation may also provide therapeutic benefits in more common diseases, where cell-specific accumulation of H_2_S has been reported. For example, a decrease in the enzymes involved in the H_2_S oxidation pathway has been reported in the gut of mouse models and patients with Crohn’s Disease, together with a relative increase in the abundance of H_2_S microbial producers, resulting in pathological accumulation of H_2_S that contributes to the disease [67], and the increase in CBS has been reported in human biopsies of precancerous adenomatous polyps [68].

The transsulfuration pathway, and specifically, mRNA levels, protein levels and activities of CSE or CBS, has been linked to alterations in other metabolic pathways, such as serine and nucleotides biosynthesis or folate cycle [7,69,70]. Several evidences have demonstrated the contribution of these alterations to pathophysiological states related to mitochondrial dysfunction, such as: (1) the inhibition of CI in human cells causes an upregulation of the mRNA levels of *CBS* and *CSE*, together with the upregulation of *PSAT1*, *SHMT2* and *PHGDH*, which are involved in serine/glycine biosynthesis and folate cycle [71], (2) the levels of the MTHF2 protein, involved in the folate cycle, are increased in the Deletor mouse model, a mouse model with accumulation of mtDNA deletions, resulting in modifications in one-carbon metabolism [72], (3) the levels of MTHF2 and SHTM2 proteins are increased in other mouse models of mitochondrial diseases caused by defects in mtDNA [7], (4) *CBS*, *CSE*, *PHGDGH*, *PSPH* and *PSAT1* mRNAs are upregulated in other human cell models of pharmacologically induced mtDNA depletion [73] and (5) *MTHFD1*L, *MTHFD2*, *PSAT1* and *PHGDH* are upregulated in patients with mitochondrial myopathy caused by mtDNA deletion [70]. Thus, the regulatory effects of CoQ over sulfide metabolism could indirectly modulate these pathways and, consequently, provide therapeutic benefits on these pathophysiological states (Figure 3). In fact, the serine/glycine ratio is increased by the supplementation with CoQ_10_ in vitro, together with a metabolic adaptation of nucleotides biosynthesis and folate cycle [64]. Therefore, CoQ_10_ supplementation could be beneficial in pathologies with alterations in sulfide metabolism, serine biosynthesis, folate cycle or nucleotide metabolism by repressing *CBS*, *CSE*, *PHGDH*, *PSAT1* and *MTHDF1L*, among others [64].

The role of CoQ in sulfide metabolism and related pathways has also been demonstrated in models of primary CoQ deficiency. Two independent studies showed that either in vitro, in skin fibroblasts from patients with primary CoQ_10_ deficiency, or in vivo, in two different mouse models of primary CoQ deficiency, the deficiency in CoQ leads to reduced activity and levels of SQOR [74,75]. The consequence of this reduction is an increase in the amount of H_2_S and a reduction in the levels of glutathione (GSH), an antioxidant composed by glutamate, cysteine and glycine, the last two linked to H_2_S metabolism [74,75]. In one study, two mouse models of CoQ deficiency caused by different mutations in *Coq9* were used [75]. The *Coq9^Q95X^* mouse model, which has a mild reduction of CoQ levels and shows mild signs of late-onset myopathy, has reduced levels and activity of SQOR in the skeletal muscle. The *Coq9^R239X^* mouse model, which has a severe reduction in CoQ levels and suffers a fatal mitochondrial encephalopathy, has severe reduction in the levels and activity of SQOR in the brain, kidneys and skeletal muscle [75], and, as a consequence, the CBS levels are increased [64]. Besides, the levels of SQOR downstream enzymes, Sulfite Oxidase (SO) and Thiosulfate Sulfurtransferase (TST), but not ETHE1, are increased, most likely as a compensatory mechanism [75]. Moreover, the most clinically affected tissue in *Coq9^R239X^* mice, the brain, shows a reduction in the levels of total GSH, together with a decrease in the levels and activities of the glutathione reductase (GRd) and glutathione peroxidase 4 (GPx4) enzymes [75]. Interestingly, CoQ and GPx4, together with the apoptosis-inducing factor mitochondria-associated 2 (AIFM2), have been related to the suppression of ferroptosis, a cellular process that has not been evaluated in CoQ deficiency [76,77]. Furthermore, the levels of specific metabolites of the serotonin biosynthesis pathway and the levels of tyrosine are increased. This may suggest that sulfide metabolism can interact and alter the neurotransmitter biosynthesis. Accordingly, similar alterations in the levels of L-glutamate, dopamine and 5-hydroxyindoleacetic acid are detected in wild-type animals treated with H_2_S donors [75]. Furthermore, the effects of H_2_S, a well-known vasodilator [78], cause low blood pressure in *Coq9^R239X^* mice. These abnormalities are rescued by the treatment with b-RA [51], which, as previously mentioned, rescues CoQ deficiency due to mutations in *Coq7* or *Coq9* [41,51,52,53].

Another study used the *Pdss2^kd/kd^* mice, which suffer from nephrotic syndrome [74]. *Pdss2^kd/kd^* mice show severe reduction in the levels of SQOR in the kidneys. In this model, however, contrary to what was observed in *Coq9* mutant mice and human CoQ-deficient fibroblasts, also, SQOR downstream enzymes TST, SO and ETHE1 are downregulated, indicating genotype- and/or tissue-specific mechanisms. As a consequence, H_2_S levels are increased, and GSH levels are decreased in the kidneys of *Pdss2* mice [74]. Moreover, urine and plasma thiosulfates are decreased, and short-chain acylcarnitine is increased in plasma of these mice. The altered acylcarnitine profile might result from inhibition of short-chain Acyl-CoA dehydrogenase (SCAD) by H_2_S, or might be the consequence of low levels of CoQ on lipid metabolism, as discussed in the next section of this review. In any case, chronic administration of oral CoQ_10_ increases SQOR and the other enzymes of the H_2_S oxidation, and GSH levels, normalizes plasma acylcarnitine profile and prevents renal failure in *Pdss2^kd/kd^* mice [48]. Therefore, the disruption in sulfide metabolism is one of the pathomechanisms of primary CoQ deficiency and it should be considered for the development of new treatments [47,51,74,75].

## 4. Other CoQ-Linked Reactions in Mitochondria

CoQ receives electrons from other enzymes of different metabolic pathways in the CoQ-junction (Figure 1). It receives electrons from dihydroorotate dehydrogenase (DHODH), which catalyzes the conversion of dihydroorotate to orotate, the fourth reaction step within pyrimidine de novo biosynthesis [79]. Therefore, the pyrimidine de novo biosynthesis is directly connected to CoQ and, consequently, low levels of CoQ may impair uridine-5′-triphosphate (UTP), cytidine 5′-triphosphate (CTP) and deoxythymidine triphosphate (dTMP) synthesis, as well as RNA and DNA synthesis. In fact, supplementation with uridine, the precursor of UTP, CTP and dTMP in the pyrimidine salvage pathway [80], increases the growth rate in CoQ_10_-deficient fibroblasts, but not in wild-type fibroblasts [36]. Moreover, secondary CoQ_10_ deficiency has been reported in patients with mitochondrial DNA depletion due to mutations in *DGUOK* (encoding mitochondrial deoxyguanosine kinase, which is involved in the purine nucleotide salvage pathway), *SUCLA2* (encoding the β subunit of succinyl-CoA synthase, which is involved in the Krebs cycle), *MPV17* (involved in mitochondrial deoxynucleoside triphosphates pool homeostasis) or from unknown etiology [81,82]. The CoQ-dependent DHODH seems to also be important in tumorigenesis since the pyrimidine biosynthesis required in mouse breast cancer cells need a functional CoQ redox-cycling and DHODH activity [83].

CoQ also receives electrons from the electron transfer flavoprotein-dehydrogenase (ETFDH), which serves as a short electron transfer pathway to conduct electrons from nine different mitochondrial flavin adenine dinucleotide (FAD)-containing acyl-CoA dehydrogenases of fatty acid β-oxidation and amino acid catabolism to the ubiquinone pool [84]. Curiously, some patients with mitochondrial myopathy due to mutations in *ETFDH* show CoQ_10_ deficiency, although the precise mechanisms for this association are unknown [85,86]. Importantly, a trend toward decreased levels of short- and medium-length acylcarnitines are showed in the liver and kidneys of *Pdss2^kd/kd^*, while the levels of acylcarnitines C4, C5 and C6 are increased in plasma of the same mice, indicating a defective oxidation of fatty acids [74].

A third type of enzymes that use CoQ as electron acceptors are proline dehydrogenase and proline dehydrogenase 2 (PRODH and PRODH2) [87,88], which are related with proline, glyoxylate and arginine metabolism. Interestingly, PRODH2 has been proposed as a molecular target for treating primary hyperoxaluria, and some CoQ analogs seem to stimulate PRODH2 activity [87]. The remaining mitochondrial enzymes that use CoQ are glycerol 3-phosphate dehydrogenase (GPD2) [89], which connects glycolysis, OxPhos and fatty acids metabolism, and choline dehydrogenase (CHDH) [90], which is involved in the glycine metabolism. Remarkably, the behavior of these CoQ-linked proteins in CoQ deficiency seems to be different from SQOR, since kidneys of *Coq9^R239X^* mice show an increase in the levels of ETFDH, CHDH, DHODH, PRODH and PRODH2 [3] (Table 1), most likely due to a compensatory mechanism, although the functional evaluation of theses enzymes has not been assessed in CoQ deficiency.

Other mitochondrial components susceptible to be regulated by redox reactions are the UCPs. Controversial results have been reported on the involvement of CoQ in the regulation of the UCPs. By using bacterial overexpressed UCP1, 2 and 3 in liposomes, Echtay and colleagues [91,92] showed that CoQ, but not CoQH_2_, activated the H+ transport through the UCPs. These studies suggested that CoQ acts as a non-covalent UCP cofactor in cooperation with other well-known UCPs modulators, such as free fatty acids or retinoids. Later on, Jaburek and Garlid [93] optimized the conditions of isolation and refolding of bacterially expressed UCPs and evaluated the effect of CoQ in the same model. Contrary to what was reported by Echtay and colleagues [91,92], they found that CoQ had no effect on the proton transport catalyzed by any of the UCPs [93]. Similar conclusions were documented by Esteves and collaborators using a CoQ-deficient yeast model [94]. Nevertheless, a more recent study showed that CoQ, through its redox state, is a regulator of the inhibition, by purine nucleotides, of free fatty acid-activated UCP1 homologues under phosphorylating respiration conditions [95]. Therefore, additional studies are required to elucidate the role of CoQ in the modulation of UCPs activities, especially under physiological conditions and taking into account potential type- and tissue-specific differences. Also, some reports have shown that CoQ_10_, as well as other short-tail CoQ analogs, can function as modulators of apoptosis by the regulation of the mitochondrial permeability transition pore (PTP) [96,97,98]. As it happens with other CoQ functions, the size of the polyprenoid tail has functional implications, i.e., CoQ_10_ inhibits PTP opening induced by H_2_O_2_, CoQ_5_ does not produce any effect on the PTP opening induced by H_2_O_2_, while CoQ_0_ induces PTP opening and H_2_O_2_ production [99]. However, cell- and tissue-specific differences have been observed [99,100], and, consequently, additional studies in physiological and pathophysiological conditions are required to better understand the role of CoQ_10_ and other short-tail analogs in the regulation of the mitochondrial PTP opening.

## 5. Conclusions and Perspectives

From the first isolation of CoQ, its role in the mitochondrial respiratory chain and energy production has been clearly demonstrated. The generation and characterization of different models of CoQ deficiency and studies of CoQ_10_ supplementation in the same models have confirmed it. Also, recent studies have unveiled the importance of the redox state of CoQ in the regulation of super-complexes’ formation and, consequently, in the use of the reducing equivalents by the respiratory chain, as well as in the production of ROS. These data could open the possibility to modulate/modify the energy metabolism through physiological or pharmacological interventions that target the redox state of CoQ. Besides its implication in the mitochondria respiratory chain, CoQ is also a key component in the reactions mediated by other mitochondrial enzymes and, therefore, CoQ links the energy production to other metabolic pathways of the cell. The contribution of these CoQ-linked enzymes to RET must be better defined in different physiological and pathophysiological conditions. Also, whether all these metabolic pathways related to the CoQ-linked enzymes are influenced by CoQ levels, including the cases of primary and secondary CoQ deficiencies, or CoQ redox state, remains to be evaluated. However, recent studies indicate that modifying the levels of CoQ and/or its redox state indeed affects some of these pathways, i.e., sulfide metabolism, in the mitochondrial and cytosolic compartments. In any case, the link between CoQ, sulfide metabolism, one-carbon metabolism, glutathione and ferroptosis shown in recent studies must be validated and further investigated in different models. Furthermore, those pathways could be influenced by an alteration in the distribution of CoQ among the different compartments of the cell. However, our knowledge about the mechanisms of CoQ distribution in the cell is still very limited, and it is unknown if the cell responds with a redistribution in the CoQ pool under CoQ deficiency. Together, the results exposed above prove the relevance of CoQ_10_ beyond its classical function as an electron carrier in the mitochondrial respiratory chain and, consequently, they may have significant implications in the use of CoQ_10_ supplementation in patients with different metabolic diseases. Nevertheless, additional research studies are required in models of CoQ deficiency, models of CoQ_10_ supplementation and models with different redox states of CoQ, paying close attention to the bioavailability of the exogenous CoQ_10_ and the potential cell- and tissue-specific differences.

## Figures and Tables

**Table 1:** Levels of the CoQ-linked proteins in *Coq9^R239X^* mice compared to *Coq9^+/+^* mice. The values are expressed as Log_2_ (Fold-Change (*Coq9^R239X^*/*Coq9^+/+^*)). Data obtained from Reference [3].

Tissue				
Protein		Kidneys	Heart	Cerebellum
	SQOR	−1.2755	−1.1292	
	GPD2	−0.0407	0.2659	0.0454
	ETFDH	0.1131	−0.1072	0.0665
	CHDH	0.1572		
	DHODH	0.2237		
	PRODH	0.4076	0.1372	0.5226
	PRODH2	0.6400		

The results were obtained by liquid chromatography (LC) - Mass spectrometry (MS)/MS using tandem mass tagging to measure relative protein abundances [3].
